# Supplementary material for: A Novel Isogenic Human Cell-Based System for MEN1 Syndrome Generated by CRISPR/Cas9 Genome Editing
Source: Int J Mol Sci. 2021 Nov 8;22(21):12054. doi: 10.3390/ijms222112054 (PMC8584395; doi:10.3390/ijms222112054)
Supplement: Supplementary file 1 [file ijms-22-12054-s001.zip › ijms-1407823-supplementary.pdf]

International Journal of *Molecular Sciences*

MDPI

Supplementary Materials

A novel isogenic human cell-based system for MEN1 syndrome generated by CRISPR/Cas9 genome editing

Natalia Klementieva, Daria Goliusova, Julia Krupinova, Vladislav Yanvarev, Alexandra Panova, Natalia Mokrysheva and Sergey L. Kiselev

Table S1. Predicted off-target sites for gRNA-MEN1.

Name	Sequence (5′-3′)	Mismatches	Locus
Wild-type	CACCTGCTGCGATTCTACGA	1	chr11:-64572606
Off-target site #1	CCCCTGCTGGGATTCTGCTA	3	chr11:+7466151
Off-target site #2	AAGCAGCTACGATTCTACTA	4	chr10:+76789648
Off-target site #3	TACATGCTGTGATTCTACTG	4	chr2:-111664537

Table S2. STR analysis results.

Line / Locus	hiPSMEN1-MNA-6	hiPSMEN1-MNA-6-e65	hiPSMEN1-MNA-6-e67	hiPSMEN1-MNA-6-e85	hiPSMEN1-MNA-6-e87
**AMEL**	X,X	X,X	X,X	X,X	X,X
**D3S1358**	15,16	15,16	15,16	15,16	15,16
**TH01**	6,6	6,6	6,6	6,6	6,6
**D12S391**	19,22	19,22	19,22	19,22	19,22
**D1S1656**	12,16	12,16	12,16	12,16	12,16
**D10S1248**	15,17	15,17	15,17	15,17	15,17
**D22S1045**	15,15	15,15	15,15	15,15	15,15
**D2S441**	11,14	11,14	11,14	11,14	11,14
**D7S820**	8,8	8,8	8,8	8,8	8,8
**D13S317**	11,11	11,11	11,11	11,11	11,11
**FGA**	19,25	19,25	19,25	19,25	19,25

**TPOX**	9,11	9,11	9,11	9,11	9,11
**D18S51**	14,15	14,15	14,15	14,15	14,15
**D16S539**	12,12	12,12	12,12	12,12	12,12
**D8S1179**	13,15	13,15	13,15	13,15	13,15
**CSF1PO**	11,12	11,12	11,12	11,12	11,12
**D5S818**	10,11	10,11	10,11	10,11	10,11
**vWA**	18,19	18,19	18,19	18,19	18,19
**D21S11**	30,30	30,30	30,30	30,30	30,30
**SE33**	17,30.2	17,30.2	17,30.2	17,30.2	17,30.2

Table S3. Antibodies and their final dilutions used for immunofluorescence analysis and flow cytometry.

**Antibody**	**Dilution**	**Catalog number, vendor**
Anti-Oct4	1:200	ab19857, Abcam
Anti-SSEA-4	1:200	MC-813-70, Thermo Fisher Scientific
Anti-Sox2	1:200	ab97952, Abcam
Anti-TRA-1-60 DyLight 488 conjugated	1:100	MA1-023-D488X, ThermoFisher
Anti-AFP	1:100	ab54745, Abcam
Anti-Vimentin	1:200	ab92547, Abcam
Ant-Cytokeratin-19	1:2000	ab52625-100, Abcam
Anti-TUBB3	1:500	801207, Biolegend
Anti-SOX17	1:1000	PA5-72815, Thermo Fisher Scientific
Anti-FOXA2	1:300	ab108422, Abcam
Anti-CXCR4	1:200	ab124824, Abcam
Anti-Menin	1:500	A300-105A, Bethyl Laboratories
Goat anti-Mouse IgG H&L, Alexa Fluor 594	1:500	ab150120, Abcam

Goat anti-Mouse IgG H&L, Alexa Fluor 488	1:500	ab150117, Abcam
Goat anti-Rabbit IgG H&L, Alexa Fluor 594	1:500	ab150084, Abcam
Goat anti-Rabbit IgG H&L, Alexa Fluor 488	1:500	ab150077, Abcam

Table S4. Oligonucleotides for generating sgRNA expression plasmid.

Name	Sequence (5′-3′)
sgRNA-MEN1 top strand	CACCTGCTGCGATTCTACTAGTTTT
sgRNA-MEN1 bottom strand	TAGTAGAATCGCAGCAGGTGCGGTG

Table S5. Primers for potential off-target effects evaluation.

Name	Forward primer (5′-3′)	Reverse primer (5′-3′)
Off-1_men1	CTGGGACTTTGCCTTTGATCTC	ATTTCTCCCTTCTGCCTCTCTC
Off-2_men1	GAAACCAGTCCCATGATGGATG	CTGACCCATTCGCTCGTATAAG
Off-3_men1	CTCAGCCTTCTTGACCTGGTAA	GCTCTGAGAAAGACCATGTGAG

Supplementary sequences

Sequences are given from left to right in the 5′ to 3′ direction.

sgRNA-MEN1:

CACCTGCTGCGATTCTACTA

Single-stranded oligodeoxynucleotide (ssODN):

AAAACGGCCTAGGGACTGCACAAGAAAGGTGGCCCAGCCCACGTGCAGCACAGGCGTGGGACTGCCCTCCT
CCCATTTGCAGATGCCGTCATAGAAGCGCAGCAGGTGGGCGAAGCACTCAGGGTCC

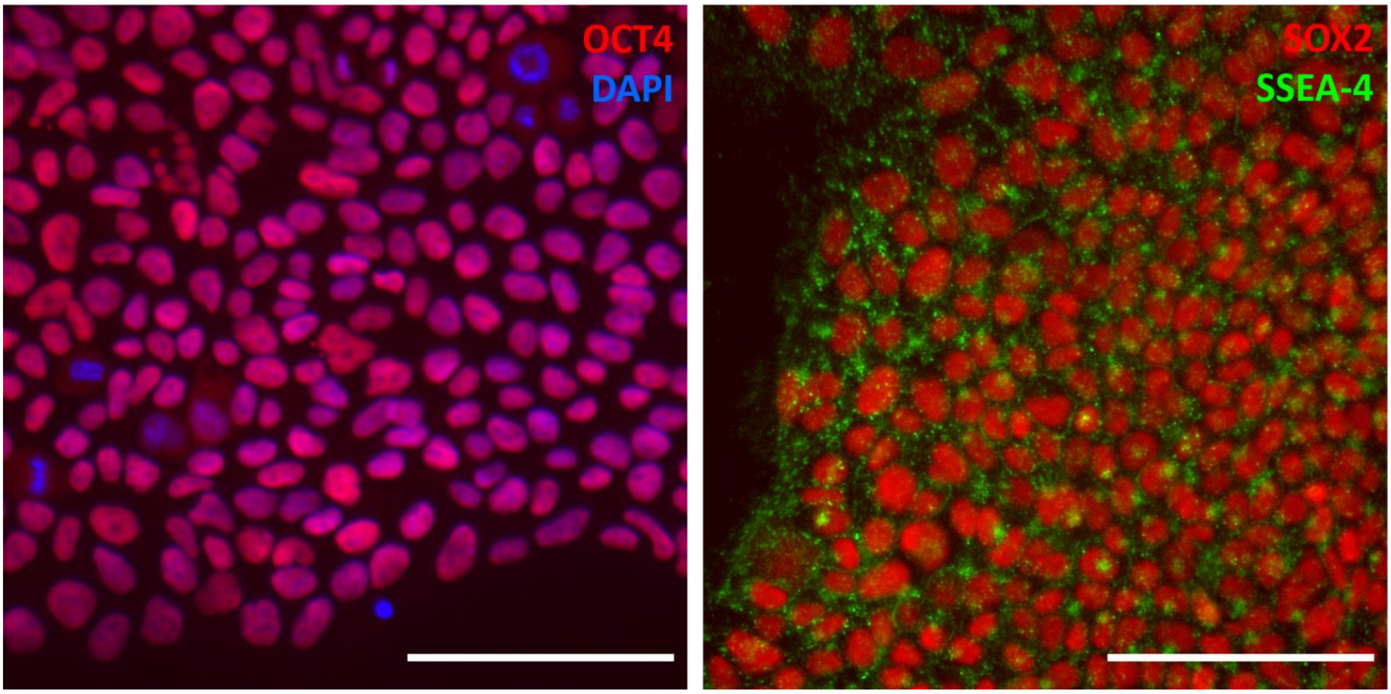

Figure S1. Positive control for immunofluorescence analysis of pluripotency. Immunostaining for OCT4, with nuclei counterstaining by DAPI (left panel), as well as for SOX2 (red) and SSEA-4 (green) (right panel) in human embryonic stem cell line hESKM-05, showing the expression of pluripotent genes. Scale bars represent 100 μm.

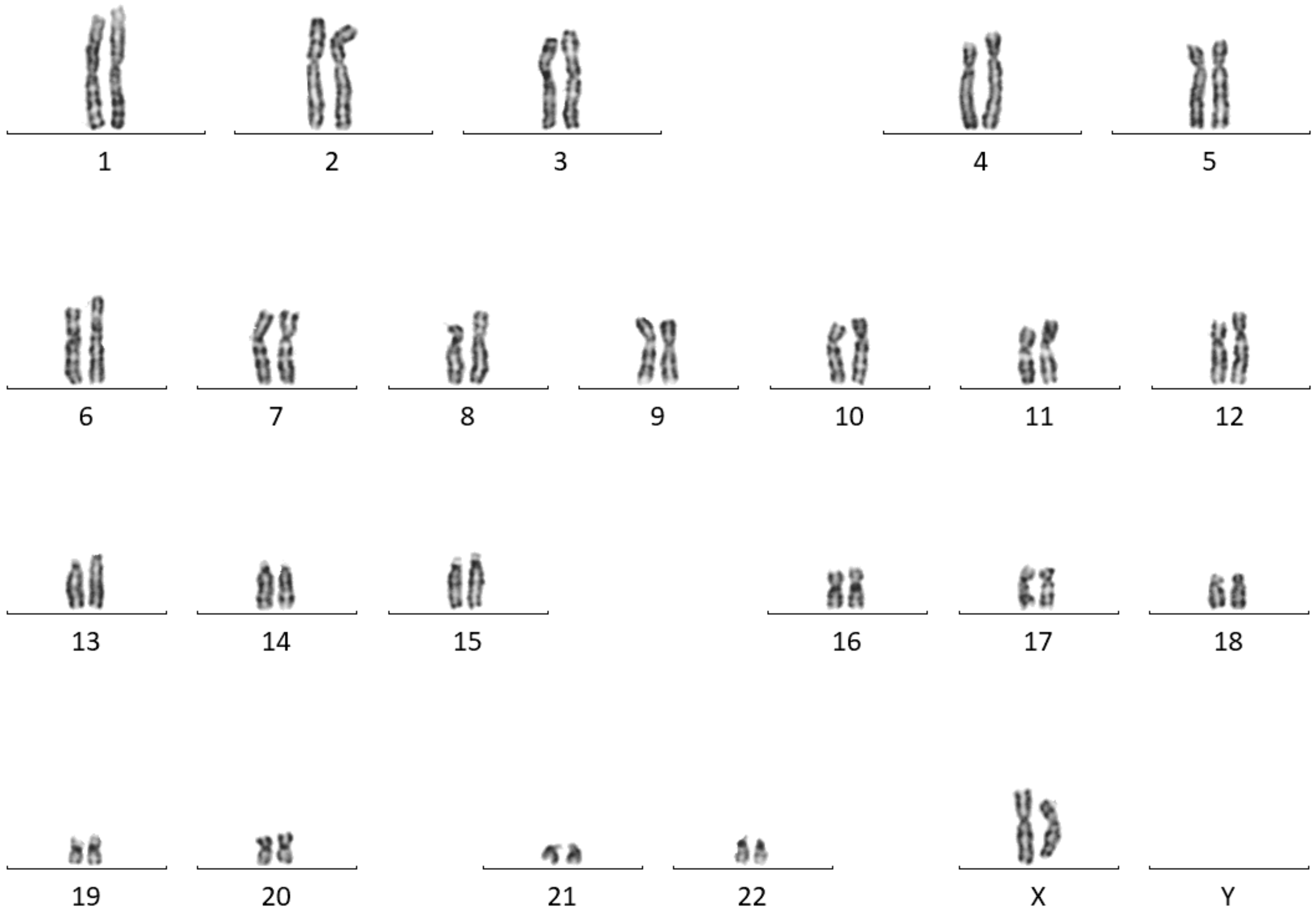

Figure S2. G-banding karyotype of the hiPSMEN1-MNA-6 cell line showing 46,XX. The analysis indicated no chromosomal abnormalities in iPSCs derived from a patient with MEN1 syndrome.

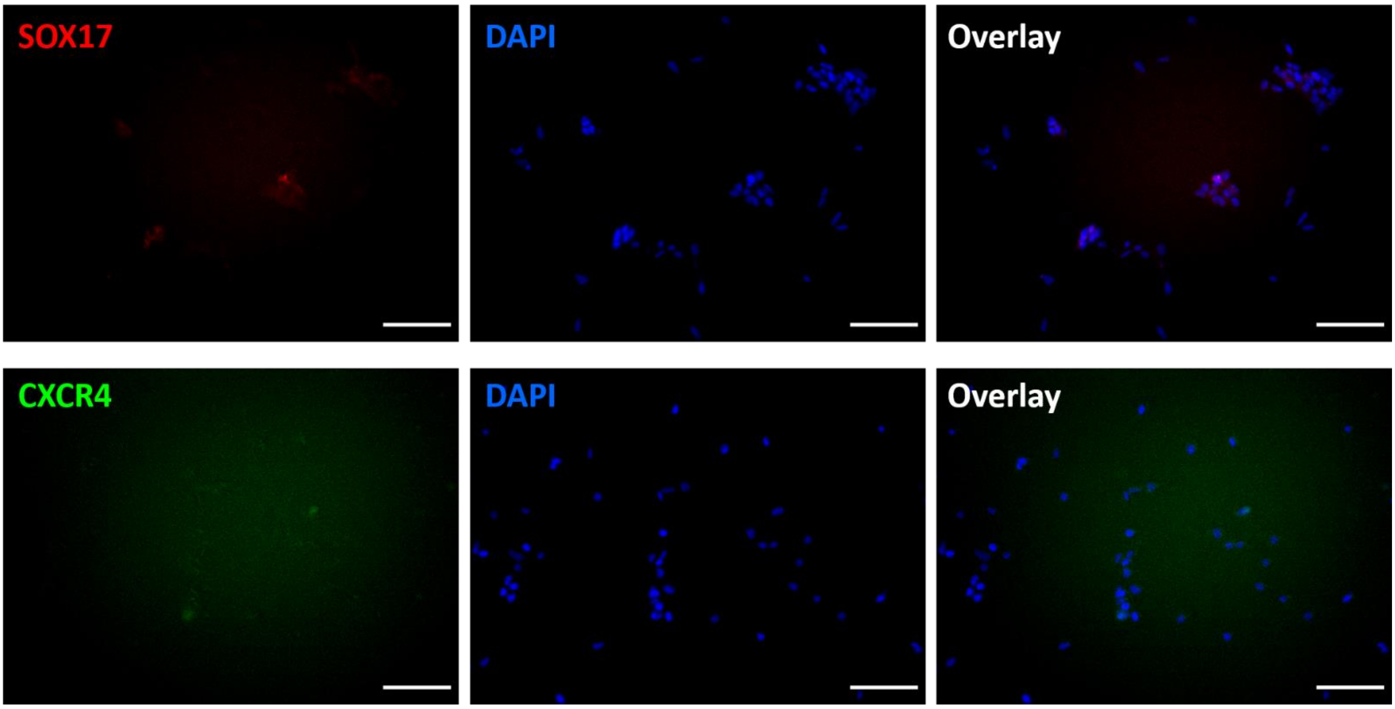

Figure S3. Negative control for immunofluorescence analysis of definitive endoderm (DE) development. The hiPS12 cells stained with anti-SOX17 and anti-CXCR4 antibodies on Day 2 of DE differentiation. Scale bars represent 100 μm. The data show no specific staining.

Differentiated H9 cells, staining for SOX17

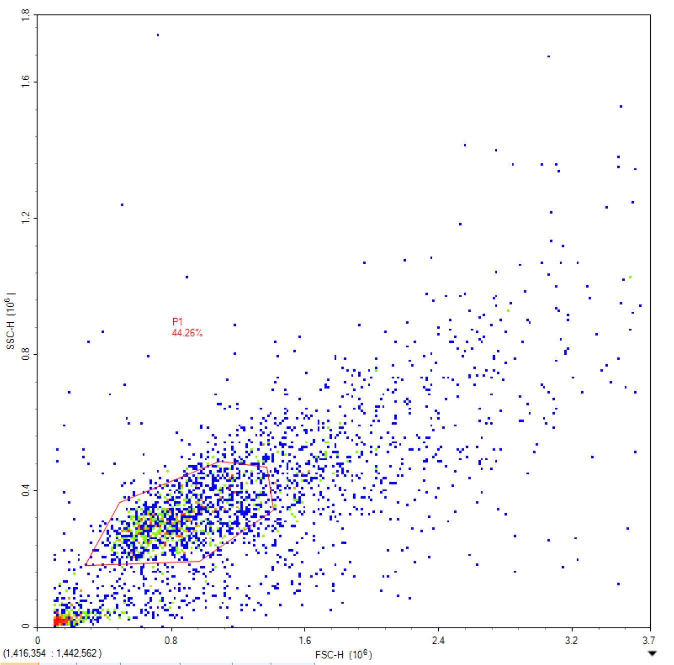

Gate	Count	% All	Mean X	Mean Y	CV X	CV Y
All	2,903	100.00 %	1,148,656.50	362,016.34	76.36 %	124.79 %
P1	1,285	44.26 %	858,472.19	317,930.59	27.72 %	21.05 %

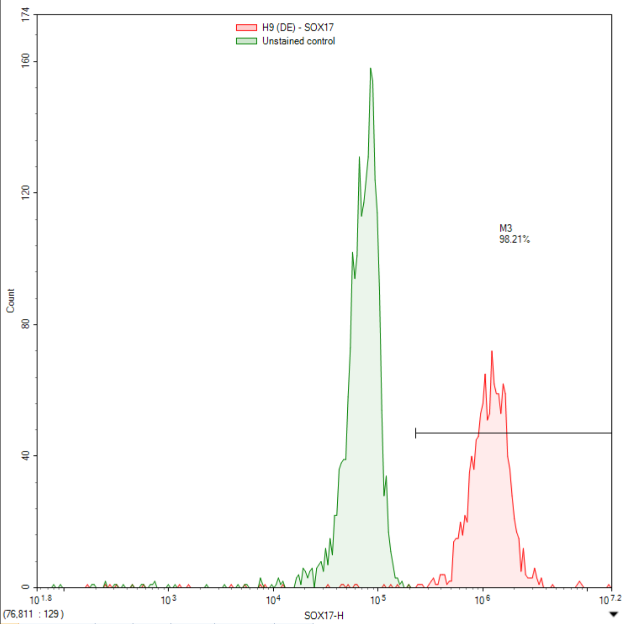

#	Sample	Gate	Count	% P1	Mean X	CV X
1	H9 (DE) - SOX17	P1	1,285	100.00 %	1,295,429.38	61.42 %
1	H9 (DE) - SOX17	M3	1,262	98.21 %	1,318,280.50	59.50 %
2	Unstained control	P1*	2,180	100.00 %	75,213.53	33.42 %
2	Unstained control	M3	0	0.00 %	0.00	0.00 %

Differentiated hiPSMEN1-MNA-6 cells, staining for SOX17

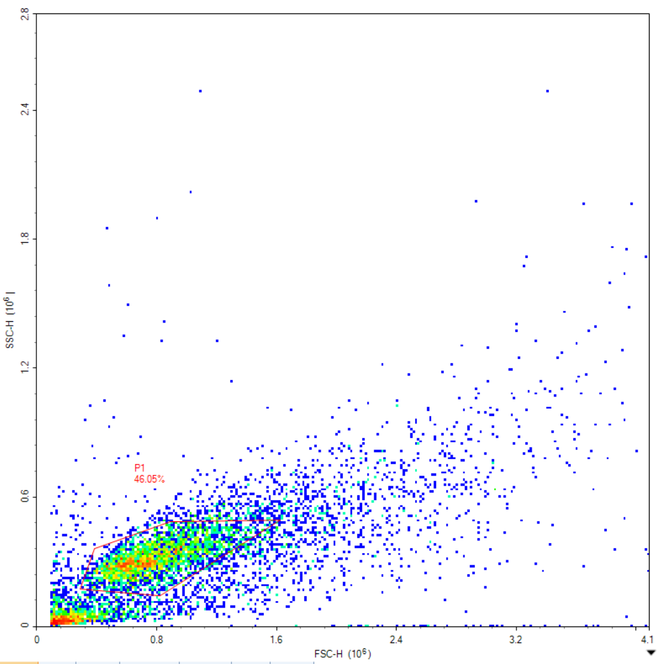

Gate	Count	% All	Mean X	Mean Y	CV X	CV Y
All	6,515	100.00 %	1,101,459.38	352,242.22	96.07 %	184.82 %
P1	3,000	46.05 %	807,500.44	319,915.34	30.94 %	23.67 %

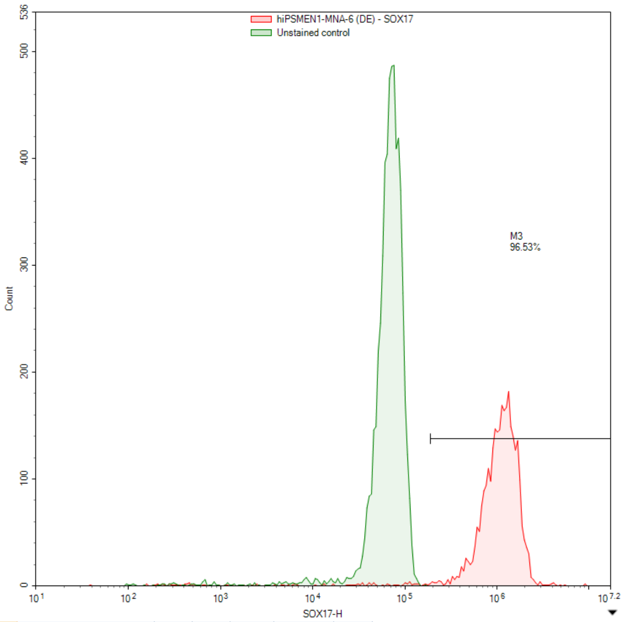

#	Sample	Gate	Count	% P1	Mean X	CV X
1	hiPSMEN1-MNA-6 (DE) - SOX17	P1	3,000	100.00 %	1,168,330.38	47.85 %
1	hiPSMEN1-MNA-6 (DE) - SOX17	M3	2,896	96.53 %	1,208,789.63	43.50 %
2	Unstained control	P1*	5,812	100.00 %	71,166.77	31.19 %
2	Unstained control	M3	0	0.00 %	0.00	0.00 %

Differentiated H9 cells, staining for CXCR4

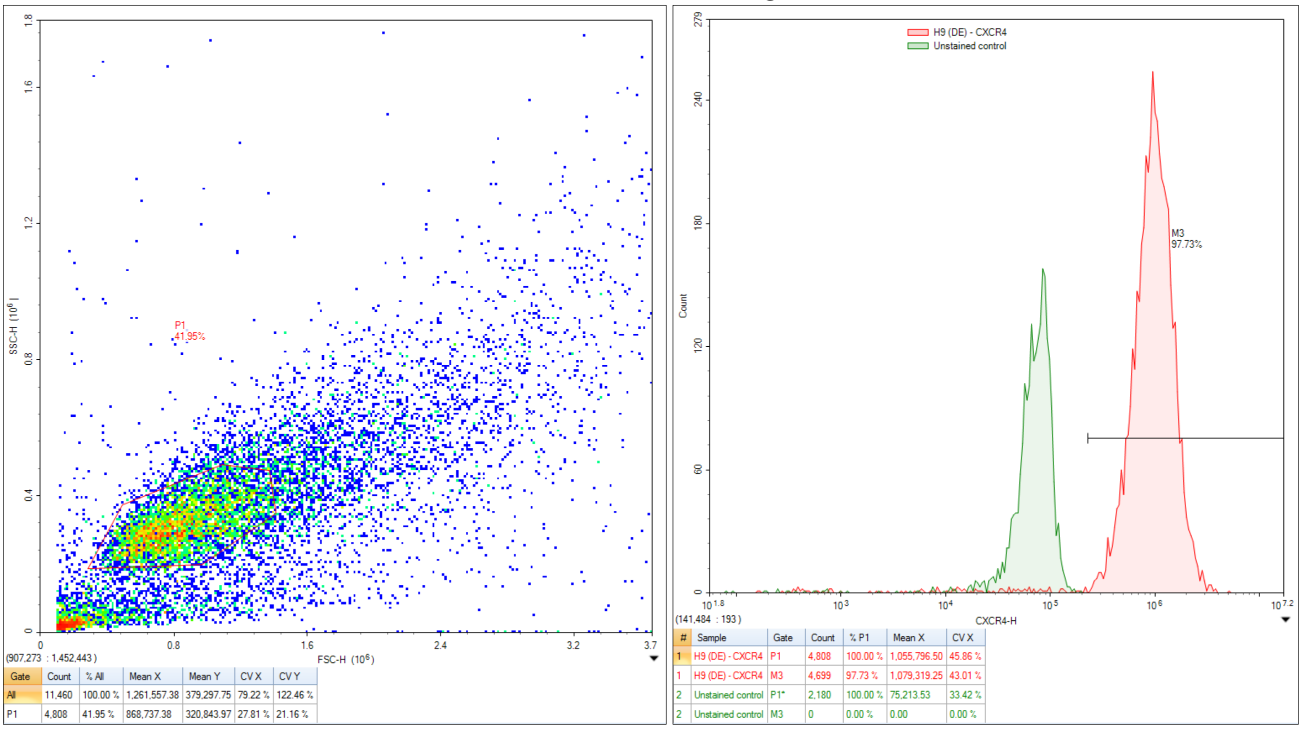

Gate	Count	% All	Mean X	Mean Y	CV X	CV Y
All	11,460	100.00 %	1,261,557.38	379,297.75	79.22 %	122.46 %
P1	4,808	41.95 %	868,737.38	320,843.97	27.81 %	21.16 %

#	Sample	Gate	Count	% P1	Mean X	CV X
1	H9 (DE) - CXCR4	P1	4,808	100.00 %	1,055,796.50	45.86 %
1	H9 (DE) - CXCR4	M3	4,699	97.73 %	1,079,319.25	43.01 %
2	Unstained control	P1*	2,180	100.00 %	75,213.53	33.42 %
2	Unstained control	M3	0	0.00 %	0.00	0.00 %

Differentiated hiPSMEN1-MNA-6 cells, staining for CXCR4

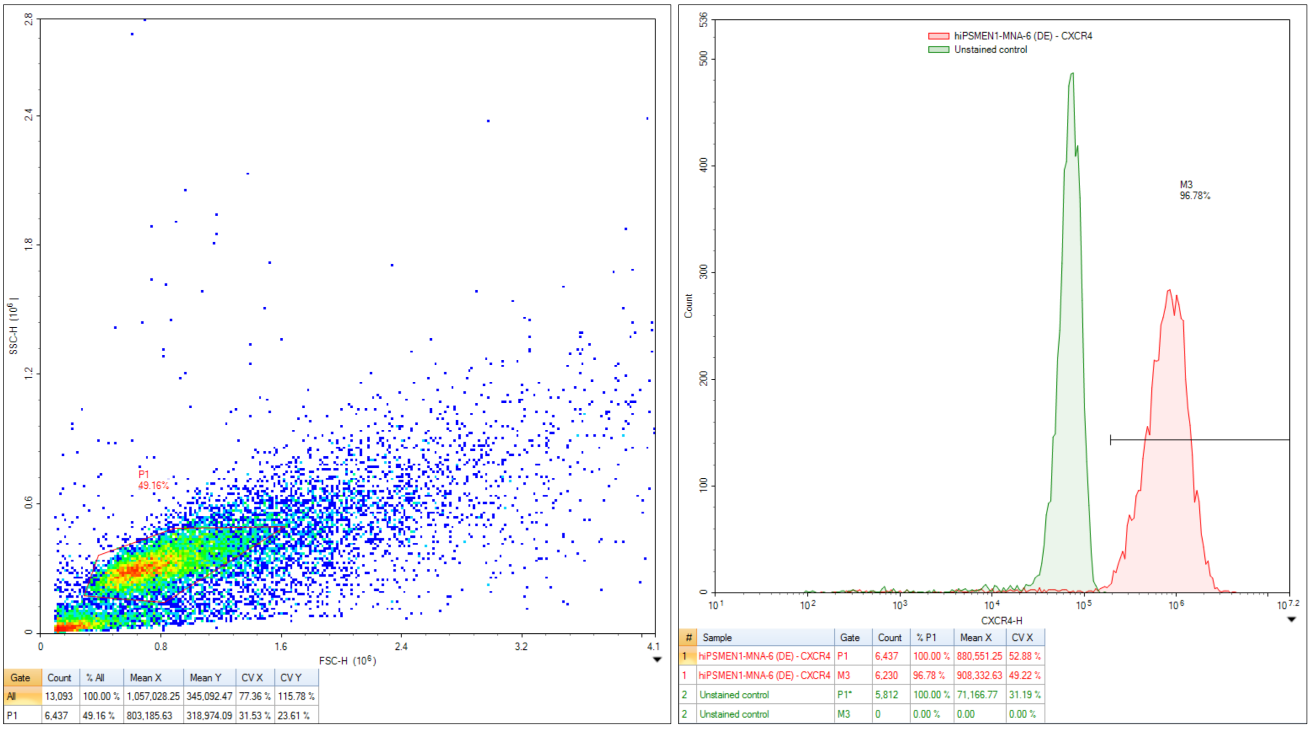

Gate	Count	% All	Mean X	Mean Y	CV X	CV Y
All	13,093	100.00 %	1,057,028.25	345,092.47	77.36 %	115.78 %
P1	6,437	49.16 %	803,185.63	318,974.09	31.53 %	23.61 %

#	Sample	Gate	Count	% P1	Mean X	CV X
1	hiPSMEN1-MNA-6 (DE) - CXCR4	P1	6,437	100.00 %	880,551.25	52.88 %
1	hiPSMEN1-MNA-6 (DE) - CXCR4	M3	6,230	96.78 %	908,332.63	49.22 %
2	Unstained control	P1*	5,812	100.00 %	71,166.77	31.19 %
2	Unstained control	M3	0	0.00 %	0.00	0.00 %

Differentiated H9 cells, staining for FOXA2

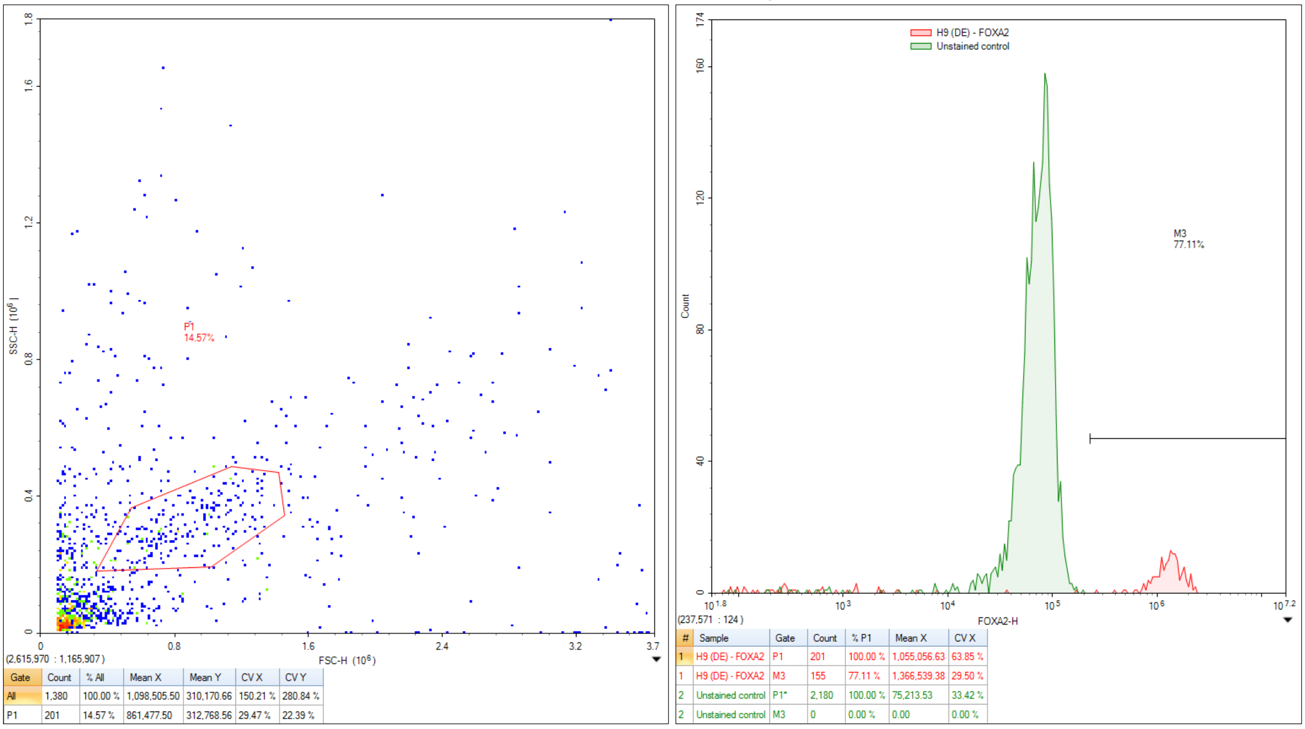

Gate	Count	% All	Mean X	Mean Y	CV X	CV Y
All	1,380	100.00 %	1,098,505.50	310,170.66	150.21 %	280.84 %
P1	201	14.57 %	861,477.50	312,768.56	29.47 %	22.39 %

#	Sample	Gate	Count	% P1	Mean X	CV X
1	H9 (DE) - FOXA2	P1	201	100.00 %	1,055,056.63	63.85 %
1	H9 (DE) - FOXA2	M3	155	77.11 %	1,366,539.38	29.50 %
2	Unstained control	P1*	2,180	100.00 %	75,213.53	33.42 %
2	Unstained control	M3	0	0.00 %	0.00	0.00 %

Differentiated hiPSMEN1-MNA-6 cells, staining for FOXA2

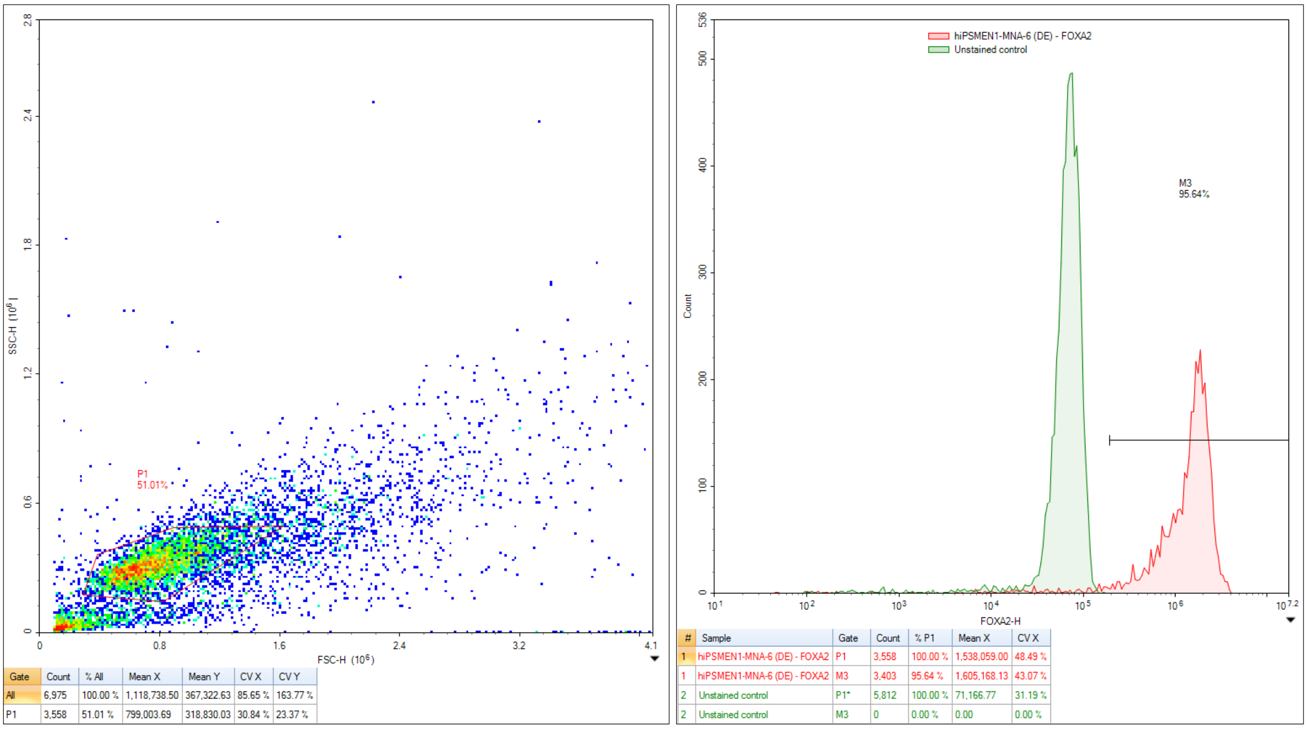

Gate	Count	% All	Mean X	Mean Y	CV X	CV Y
All	6,975	100.00 %	1,118,738.50	367,322.63	85.65 %	163.77 %
P1	3,558	51.01 %	799,003.69	318,830.03	30.84 %	23.37 %

#	Sample	Gate	Count	% P1	Mean X	CV X
1	hiPSMEN1-MNA-6 (DE) - FOXA2	P1	3,558	100.00 %	1,538,059.00	48.49 %
1	hiPSMEN1-MNA-6 (DE) - FOXA2	M3	3,403	95.64 %	1,605,168.13	43.07 %
2	Unstained control	P1*	5,812	100.00 %	71,166.77	31.19 %
2	Unstained control	M3	0	0.00 %	0.00	0.00 %

Figure S4. Flow cytometry analysis of definitive endoderm (DE) development. The data show the gating of initial cell population on dot plots of forward scatter versus side scatter (left panel) and histogram plots (right panel) presenting positive expression of DE markers SOX17, CXCR4, and FOXA2 in differentiated H9 or hiPSMEN1-MNA-6 cells (shown in red). The horizontal marker was set based on background fluorescence from unstained cells (shown in green on the histogram).

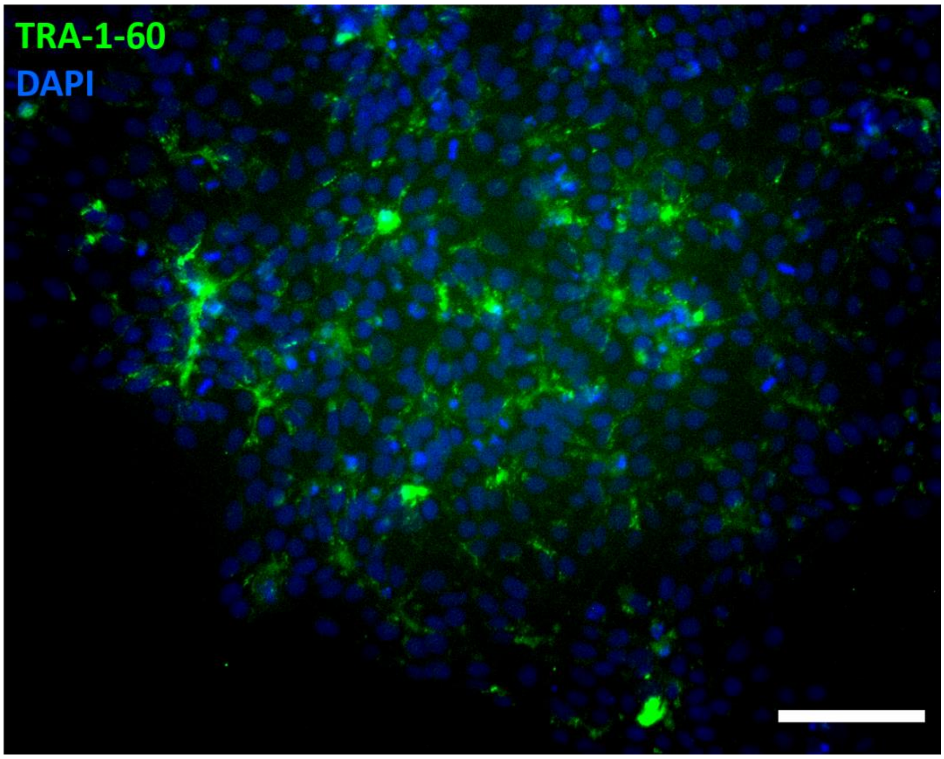

Figure S5. Immunostaining for pluripotency surface antigen TRA-1-60 in gene-corrected isogenic control cell line hiPSMEN1-MNA-6 e87. TRA-1-60 expression is shown in green and nuclei counterstaining with DAPI is shown in blue. Scale bar represents 100 μm.
